# Coconut Water: A Sports Drink Alternative?

**DOI:** 10.3390/sports11090183

**Published:** 2023-09-14

**Authors:** Brendan J. O’Brien, Leo R. Bell, Declan Hennessy, Joshua Denham, Carl D. Paton

**Affiliations:** 1 Institute of Health and Wellbeing, Federation University Australia, Mount Helen, VIC 3350, Australia; lr.bell@federation.edu.au; 2 School of Exercise and Nutrition Sciences, Faculty of Health, Deakin University, Burwood, VIC 3125, Australia; declan.hennessy@deakin.edu.au; 3 School of Health and Medical Sciences, University of Southern Queensland, Toowoomba, QLD 4300, Australia; josh.denham@usq.edu.au; 4 Centre for Health Research, Toowoomba, QLD 4300, Australia; 5 School of Health and Sport Science, Te Pukenga, The Eastern Institute of Technology, 501 Gloucester Street, Napier 4112, New Zealand; cpaton@eit.ac.nz

**Keywords:** hydration beverage, potassium, glucose, cycling, endurance performance

## Abstract

Coconut water is used as an alternative to conventional sports drinks for hydration during endurance cycling; however, evidence supporting its use is limited. This study determined if drinking coconut water compared to a sports drink altered cycling performance and physiology. In a randomized crossover trial, 19 experienced male (*n* = 15) and female (*n* = 4) cyclists (age 30 ± 9 years, body mass 79 ± 11 kg, $\dot{V}$O_2 peak_ 55 ± 8 mL·kg^−1^·min^−1^) completed two experimental trials, consuming either a commercially available sports drink or iso-calorific coconut water during 90 min of sub-maximal cycling at 70% of their peak power output, followed by a simulated, variable gradient, 20 km time trial. Blood glucose, lactate, sweat loss, and heart rate were monitored throughout the 90 min of sub-maximal cycling, as well as the time trial performance (seconds) and average power (watts). A repeated measures analysis of variance and effect sizes (Cohen’s d) analysis were applied. There were no significant differences (*p* ≥ 0.05) between the treatments for any of the measured physiological or performance variables. Additionally, the effect size analysis showed only trivial (d ≤ 0.2) differences between the treatments for all the measured variables, except blood glucose, which was lower in the coconut water trial compared to the sports drink trial (d = 0.31). Consuming coconut water had a similar effect on the cycling time trial performance and the physiological responses to consuming a commercially available sports drink.

## 1. Introduction

Prolonged endurance exercise can result in clinically significant changes in body water content and electrolyte concentration [1]. The formation of sweat to aid thermoregulation results in substantive body fluid losses. Triathletes competing in an iron man event (3.8 km swim, 180 km cycle ride, and 42.2 km run) that lasts longer than 12 h may lose from 7 to 8.5 kg of water (11–12% of body mass) [2]. Athletes may also lose a substantial amount of sodium, typically from 1 to 1.5 g per litre of sweat [3]. Subsequently, triathletes may potentially lose 12 g of sodium in an iron man event. Body water and sodium loss compromises the cardiovascular system’s ability to maintain exercise performance [4]. Furthermore, since sweat is typically hypotonic with respect to plasma, exercise-associated sweat losses lead to hypertonic hypovolemia. Reduced plasma volume is associated with increased cardio-vascular strain, a heightened perception of effort, and a decrease in muscle blood flow. [5] Weight losses of magnitudes greater than 4% of body mass result in many athletes suffering adverse conditions, including hyponatremic encephalopathy [6]. Consequently, maintaining hydration is an essential consideration for endurance athletes [7]. However, how athletes should approach hydration strategies for their event is still a matter of conjecture. 

Numerous commercial companies produce beverages explicitly formulated for athletes based on the addition of sodium. However, the impact of the depletion of other key electrolytes, such as potassium, magnesium, and calcium, on endurance performance is unclear. These electrolytes play important roles in neural transmission and muscle function [8]. For instance, disturbances in the sodium and potassium sarcolemma gradient from fluid loss can impede the propagation of action potentials from the sarcolemma to the terminal cisternae of the sarcoplasmic reticulum. This, in turn, reduces the prompt release of calcium required for muscle contractility, which may adversely affect sustained endurance performance. Potassium is the chief regulator of intracellular fluid volume. Potassium accompanies sodium excretion in sweat. Whilst potassium loss is less than sodium (approximately 6 v 40 mmol.L^−1^ [3]), the previous literature has rarely addressed the relative importance of maintaining potassium concentration on performance. 

Beverages formulated from derivatives of coconut juice naturally contain high amounts of potassium, yet the efficacy of such beverages for sustaining endurance exercise performance is limited [9,10,11]. Peart, Hensby, and Shaw [9] found no difference (*p* > 0.05) between coconut water and plain water during cycling 10 km time trial performance following 60 min of sub-maximal cycling. Kalman, Feldman, Krieger, and Bloomder [10] also found no statistical differences between coconut water (12.3 ± 5.8 min) or coconut water concentrate (11.9 ± 6.0 min), bottled water (11.9 ± 5.9 min), and a carbohydrate-electrolyte sports drink (12.8 ± 4.9 min) on time to exhaustion on a treadmill. Contrastingly, Laitano, Trangmar, Marins, Menezes, and Reis [11] observed a greater time to exhaustion in steady-state cycling (*p* = 0.029) after the ingestion of coconut water compared to plain water and a flavoured drink; however, they failed to report the magnitude of the mean differences. Coconut water alone has relatively low carbohydrate and sodium contents, so the quantity of these ingredients may need to be enhanced to provide an ergogenic effect. Therefore, this study aims to determine if consuming coconut water supplemented with carbohydrates and sodium to match a commercially available sports drink offers any additional performance benefits during endurance cycling. The main hypothesis for this study is that there will be no significant difference in terms of performance or physiological measures between the drinks.

## 2. Materials and Methods

### 2.1. Study Design

This study was a randomised crossover trial, during which the participants completed two exercise trials while consuming two different beverages: a commercial sports drink and a coconut-water-based beverage augmented with sodium and carbohydrates to match the sports drink. The commercial sports drink was chosen over water as the control condition, as this is the recognised current best practice for athletes. The trials were completed one week apart.

### 2.2. Participants

Twenty-three experienced cyclists commenced this study, and nineteen (male *n* = 15 and female *n* = 4) completed both trials (age 30 ± 9 years, body mass 79 ± 11 kg, $\dot{V}$O_2 peak_ 55 ± 8 mL·kg^−1^·min^−1^). Four participants dropped out of the study for reasons unrelated to the study. The cyclists were recruited via contact with the presidents of the region’s cycling clubs. All the cyclists gave their written informed consent to participate in the study, which was previously approved by the participating universities’ human research ethics committees in accordance with the Declaration of Helsinki. The cyclists were well-trained, with a minimum of three years of competitive experience at grade A or B (Oceania amateur grading). The study was approved by the University’s Human Research Ethics Committee before its commencement (A15-004). 

### 2.3. Pre-Test

The cyclists initially attended the sports physiology laboratory at the University for anthropometrical and physiological profiling and completed a familiarisation of the performance measures to be used in the study. The participants then completed an incremental exercise test on a cycling ergometer (Velotron, RacerMate Inc., Seattle, WA, USA), adjusted to replicate the cycling position of the participant’s own bicycle. During the incremental exercise test, their expired air was measured using a metabolic system (Moxus, AEI Technologies, Bastrop, TX, USA) for a gas (O_2_ and CO_2_) analysis. The metabolic system was calibrated before each test using alpha gas of a known composition (20.9 and 16% O_2_ and 0.3 and 4% CO_2_) and ventilation with a 3 L syringe. The incremental exercise test commenced at 150 watts, and the intensity was increased by 25 watts every second min until volitional exhaustion. The final completed 2 min wattage stage was determined from the final completed stage plus the proportion of any uncompleted stage. The peak oxygen peak ($\dot{V}$O_peak_) was determined as the highest 60 s O_2_ uptake value achieved at any point. All the laboratory testing was performed after overnight fasting at the same time of day (8–10 AM). The participants were encouraged to hydrate the night before and the morning of the testing. 

### 2.4. Experimental Trials

The participants completed two experimental trials, during which, in a randomised sequence, they consumed either a commercially available sports drink or coconut water. The administration of the beverage was single-blinded to the participant. The composition of the coconut water contained 1420 mg·L^−1^ of potassium, 448 mg·L^−1^ of sodium, and 66 g·L^−1^ of carbohydrate (1164 Kj). The sports drink contained 132 mg·L^−1^ of potassium, 458 mg·L^−1^ of sodium, and 55 g·L^−1^ of carbohydrate (970 Kj). Each trial comprised 90 min of pre-load cycling to simulate a typical long training session or competitive event. Five minutes later, the participants completed a variable gradient, self-paced, 20 km time trial on the Velotron cycle ergometer (Racermate™, Seattle, WA, USA). Immediately before cycling, the participants’ nude body mass (underwear remained on) was determined (Wedderburn scales, Japan, data recorded to ±5 g). During the pre-load exercise, the participants cycled at an intensity equivalent to 65–70% of the peak power output they achieved during the incremental exercise test, with set periods (5 min) of higher-intensity exercise at 95% of their peak power output at 15, 35, 45, 65, and 75 min. After each high-intensity interval, the power output was decreased to 40–50% for 5 min before returning to 65–70%. Five minutes after the completion of the pre-load cycling exercise, the participants immediately began a simulated, variable gradient, self-paced, 20 km time trial, which they were requested to finish as quickly as possible. Their heart rate, blood lactate, and glucose were recorded at the 30-, 60-, and 85 min marks of the pre-load exercise. Heart rate was determined via a wearable chest strap (Polar, Jyväskylä, Finland). Blood lactate (Lactate Pro2, Lactate Pro, Japan) and glucose (ACC-Check, Roche Diagnostics Switzerland, Basel, Switzerland) were analysed after the participant’s fingers were pricked with a sterile disposable lancet. Upon the completion of the 90 min pre-load, the participant’s body mass was re-measured to determine their sweat loss. Sweat loss was determined as the difference in body mass before and after the 90 min of cycling. Body mass was determined after removing clothes (except underwear) and sweat with a towel.

### 2.5. Beverage Treatment

**Figure 1:**
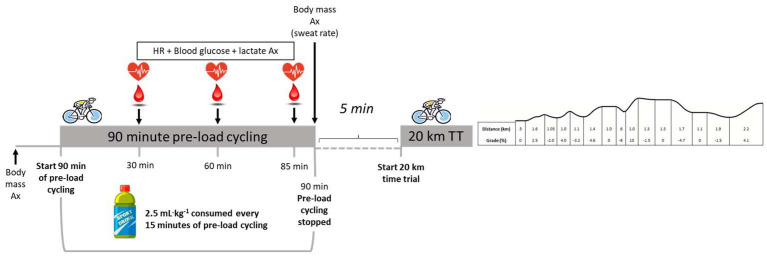
This image shows the structure and sequence of the sports drink and coconut beverage trials.

For each 15 min segment of the pre-load exercise (e.g., 15–30, 30–45, 45–60, 60–75, and 75–90 min), the participants drank 2.5 mL of fluid per kilogram of body mass of the experimental beverage. For the initial 20 km time trial, the cyclists consumed water ad libitum and replicated this amount in the subsequent trial. The trial protocol is shown in Figure 1.

### 2.6. Data Analysis

All the data are reported as means (±SD). The between-treatment differences were determined by a one-way analysis of variance (ANOVA) using the SPSS Statistics software (Version 24). The data were checked for normality via the Kolmogorov–Smirnov Test and all values were *p* > 0.05 and, therefore, were normally distributed. Where any differences between treatments were observed, an appropriate post hoc analysis with Bonferroni correction was performed. Differences were considered significant at alpha *p* < 0.05. In addition, the mean percent effects of the treatments and their 90% confidence limits were assessed using a made-for-purpose spreadsheet [12]. Two subjects revealed aberrant blood lactate concentration results (>20 mmol·L^−1^) and were excluded from the blood lactate analysis. The spreadsheet also provided the effect size statistics (*d*), which were interpreted as 0.2–0.5 being small, 0.5–0.8 being moderate, and 0.8+ being large, according to Cohen’s recommendations. Effects of <0.2 were deemed to be trivial [13]. 

## 3. Results

The means (±SD) for all the measured physiological and performance variables between each trial are shown in Table 1. There were no significant differences between the treatments (*p* > 0.05) for any of the measured variables. In addition, the effect size analysis showed only trivial differences between the treatments for all the variables, except blood glucose, which showed a small (d = 0.31) difference in the coconut water trial compared to the sports drink trial. 

**Figure 2:**
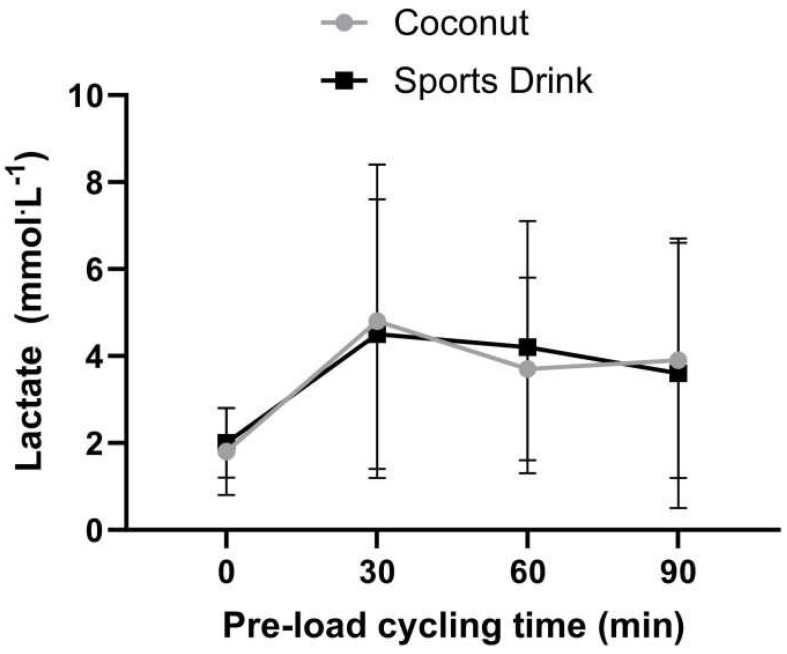
Blood lactate concentrations over the 90 min pre-load exercise task between the coconut and sports drink trials. Data are expressed as mean ± SD. A table of the preload physiological variable data is provided as a Supplementary File.

**Figure 3:**
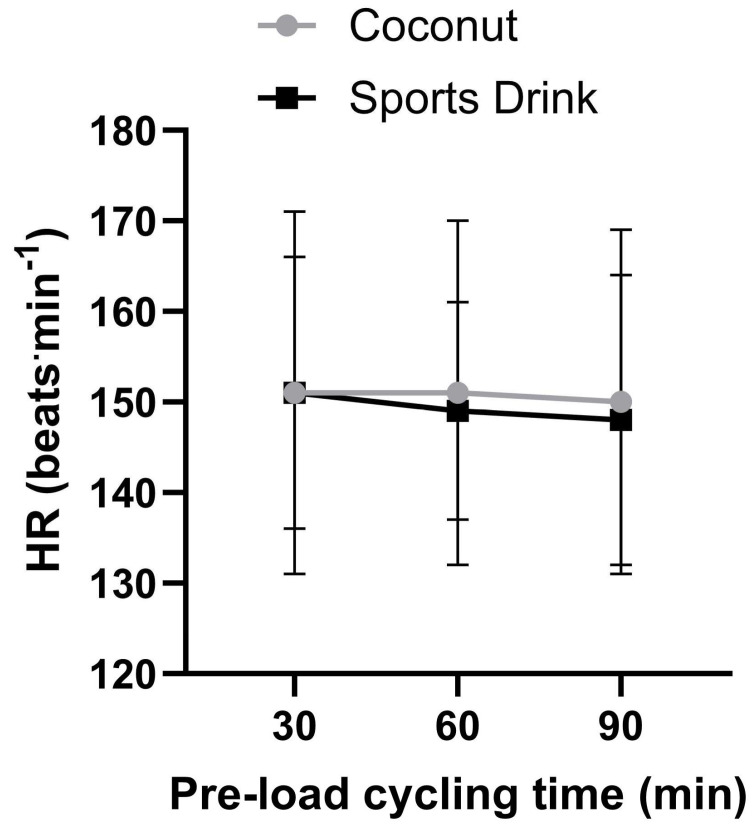
Heart rate response over the 90 min pre-load exercise task between the coconut water and sports drink trials. Data are expressed as mean ± SD. A table of the preload physiological variable data is provided as a Supplementary File.

**Figure 4:**
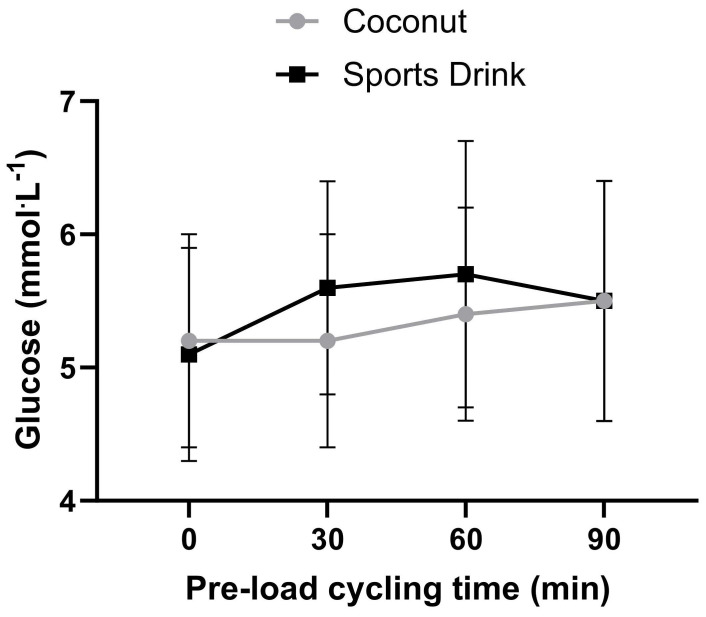
Blood glucose concentration over the 90 min pre-load exercise task between the coconut water and sports drink trials. Data are expressed as mean ± SD.

Figure 2, Figure 3 and Figure 4 show the time course response for blood lactate, heart rate, and blood glucose for both experimental trials over the 90 min steady-state exercise period. There were no significant (*p* > 0.05) differences in any of the physiological responses between the treatments. A table of the preload physiological variable data is provided as a Supplementary File.

## 4. Discussion

This study demonstrated no significant differences in the performance or physiological measures between a commercially available sports drink and coconut water during prolonged endurance cycling. Furthermore, the observed differences in heart rate, lactate, and sweat loss were trivial and those in glucose concentration were small during 90 min of high-intensity cycling. 

Previous research investigating the effects of coconut water on endurance cycling performance is limited. Laitano, Trangmar, Marins, Menezes, and Reis [11] examined the effects of consuming coconut water before a time-to-exhaustion test in hot conditions (~32°). The participants proceeded cycling at 60% of their maximal power output, maintaining a steady cadence between 60 and 70 rpm. The test was halted when the participants could no longer hold a cadence above 60 rpm and when the ratings of perceived exertion were close to 20 (Borg scale). The study found that drinking coconut water significantly improved the exercise time to exhaustion compared to plain water and flavoured beverages. However, the magnitude of these improvements was not reported. In another study, Peart, Hensby, and Shaw [9] compared the effects of consuming plain water or coconut water during 60 min of sub-maximal cycling (45–65% maximal min power) on physiological measures and subsequent 10 km time trial performance. Peart, Hensby, and Shaw [9] reported no significant differences in body mass, blood glucose, lactate, heart rate, ratings of perceived exertion, or subsequent time trial performance between coconut water and plain water (971.4 ± 50.5 and 966.6 ± 44.8 s, respectively). However, the probability of this experiment showing coconut water had an ergogenic effect compared to water was low. The duration and intensity of the exercise before the time trial were most likely insufficient to impact endogenous carbohydrate stores, fluid volume, and subsequent 10 km time trial performance.

Both hydration strategies largely mitigated the loss of body fluid. The sweat loss was 1.2 and 1.3 L in the coconut water and sports drink trials, respectively, but, based on the mean body mass of 80 kg, 1 litre of fluid on average. Consequently, the net fluid deficit after 90 min of cycling was limited to a modest 0.2 to 0.3 L on average. It is hypothesized that coconut water may have had a potential ergogenic effect, as it contains considerably more potassium than commercial sports beverages. Potassium is the principal cation of intracellular fluid, and its cellular content regulates cells’ tonicity. Based on the average participant body mass of 80 kg and the coconut water containing 1420 mg of potassium per litre, the total amount of potassium ingested in the coconut water trial was approximately 1420 mg. The potassium ingested in the sports drink trial was approximately 132 mg based on it containing 132 mg of potassium per litre. On the assumption that potassium concentration in sweat is approximately 5 mmol·L^−1^ and the potassium excretion in sweat was 200 mg·L^−1^ it is estimated potassium loss in both trials was approximately 200 mg. Therefore, the overall difference in potassium intake relative to excretion in sweat in the coconut water trial after 90 min of cycling was plus 1220 mg. Consequently, the participants gained potassium over the 90 min of pre-load exercise. In the sports drink trial, the body potassium deficit after 90 min of cycling was −68 mg. This study showed that neither a surplus of 1220 mg nor a deficiency of −68 mg impacted the 20 km time trial performance differently. Unfortunately, blood samples were not taken to determine the impact of the beverages on the blood electrolyte concentration. However, this study may be the first to show that a 4–5-fold surplus in potassium intake, relative to excretion, had no discernible performance effects. However, for participant safety, it would be prudent to monitor for clinically relevant hyperkalaemia in future research. 

The amount of sodium ingested in the sports drink trial was 458 mg. Similarly, the amount of sodium ingested in the coconut water trial was 448 mg. The sodium excreted in sweat is approximately 1000 mg·L^−1^ (sweat 40 mmol·L^−1^), although there is individual variation in sweat sodium concentration. On the assumption that the sweat composition was 40 mmol·L^−1^ [3], the net deficit of approximately 550 mg of sodium was similar between the treatments. The impact of the sodium deficit on the time trial performance could not be determined, as no pre-load control (no drink or water-only trial) was implemented in this study. However, ingesting sodium during exercise is generally not required to maintain performance for endurance events under 2 h. Sodium ingestion appears only important for “salty sweaters” (>40 mmol·L^−1^) in endurance events lasting several hours [14]. On average, the energy provided in the coconut water trial was greater than the sports drink trial 1164 versus 970 KJ. However, it appeared the 16% energy provision difference had no meaningful impact on the time trial performance.

## 5. Limitations and Future Directions

This study’s limitations included its low sample size, the calorie intakes of both treatments not being precisely matched, the pre-load exercise duration protocol not inducing sufficient dehydration, and the blood potassium and sodium concentration changes not being assessed to highlight the impact of the beverages. Future studies could investigate if a more arduous pre-load protocol that causes a greater loss of sweat and fluid deficit exposes the potential differences in the capability of high- and low-potassium beverages to impact physiology and performance. However, despite their considerable cycling training backgrounds, the participants in this study found the pre-load exercise intensity at 70% of their peak power output, interspersed with 5 min intervals at 95% and a subsequent 20 km time trial, to be particularly arduous. The lactate concentration values were approximately 4 mmol·L^−1^ after the 90 min pre-load trials in both treatments. Therefore, most cyclists were exercising close to their anaerobic threshold. Furthermore, the approximate 1.2% of body mass (1 L of water), 550 mg of sodium, and 68 mg of potassium deficit (in the sports drink trial) were most likely insufficient to impact the subsequent time trial performance. Future projects should reduce the intensity of this exercise to ensure that the participants are below their anaerobic threshold to sustain a sufficient duration of exercise that would induce a physiologically impactful water loss. The intensity of the exercise in future research could be set at a percentage of the lactate threshold or the rating of perceived exertion, rather than a percentage of the peak power output, in order to reduce the exercise strain. Furthermore, the individual variation in physiological responses is reduced if the exercise intensity is fixed to a critical power or metabolic threshold [15]. The probability of dehydration impacting performance is greater if the magnitude of body mass loss is greater than 4% [16]. The pre-load exercise task in future research may need to exceed approximately 4 h to induce sufficient water, sodium, and potassium losses to impact time trial performance. This study did not precisely match the calorie contents of both trials equally, and future projects should match these calorie contents to ensure that the energy provision does not bias the findings. 

In conclusion, this study showed that consuming carbohydrate-enriched coconut water during endurance cycling had a similar effect on physiological responses and time trial performance to consuming a commercially available sports drink.

## Figures and Tables

**Table 1:** Mean (±SD) values for the measured physiological and performance variables along with difference effects reported as significance values and effect sizes.

	Coconut Water	Sports Drink	Mean % Difference ±90% CL	ANOVA *p*-Value	Effect Size (d)	Qualitative Inference
**Physiological Variables**						
**Glucose (mmol·L^−1^)**	5.5 ± 0.5	5.3 ± 0.6	−3.0 ± 3.2	0.29	0.31	Small
**Lactate (mmol·L^−1^)**	4.22 ± 2.2	4.04 ± 2.75	4.2 ± 31.0	0.81	0.17	Trivial
**Sweat loss (L)**	1.2 ± 0.4	1.3 ± 0.5	−5.5 ± 16.6	0.37	0.12	Trivial
**Heart rate (b·min^−1^)**	151 ± 19	151 ± 13	0.5 ± 2.6	0.77	0.04	Trivial
**Performance Variables**						
**TT time** **(s)**	2863 ± 467	2892 ± 477	−1.0 ± 2.9	0.83	0.06	Trivial
**TT power (W)**	208 ± 58	204 ± 60	2.1 ± 4.9	0.79	0.07	Trivial

## Data Availability

Data will be made available per request to the corresponding author.

## Supplementary Materials

The following supporting information can be downloaded at: https://www.mdpi.com/article/10.3390/sports11090183/s1. Table S1: preload physiological variable data.
