# Supplementary material for: Coconut Water: A Sports Drink Alternative?
Source: Sports (Basel). 2023 Sep 14;11(9):183. doi: 10.3390/sports11090183 (PMC10534364; doi:10.3390/sports11090183)
Supplement: Supplementary file 1 [file sports-11-00183-s001.zip › sports-2578227-supplementary.pdf]

Supplementary Table S1. Preload physiological variable data.

		Before preload	30 min	60 min	85 min
**Sports Drink**	**Heart rate (b·min^{-1})**		151 ± 15	149 ± 12	148 ± 16
	**Blood lactate (mmol·L^{-1})**	2.0 ± 0.8	4.5 ± 3.1	4.2 ± 2.9	3.6 ± 3.1
	**Blood glucose (mmol·L^{-1})**	5.1 ± 0.8	5.6 ± 1	5.7 ± 0.9	5.5 ± 0.8
**Coconut drink**	**Heart rate (b·min^{-1})**		151 ± 20	151 ± 19	150 ± 19
	**Blood lactate (mmol·L^{-1})**	1.8 ± 1	4.8 ± 3.6	3.7 ± 2.1	3.9 ± 2.7
	**Blood Glucose (mmol·L^{-1})**	5.2 ± 0.8	5.2 ± 0.8	5.4 ± 0.9	5.5 ± 0.8
